# Quantitative Analysis of the Enhanced Permeation and Retention (EPR) Effect

**DOI:** 10.1371/journal.pone.0123461

**Published:** 2015-05-04

**Authors:** Andrew D. Wong, Mao Ye, Martin B. Ulmschneider, Peter C. Searson

**Affiliations:** 1 Department of Materials Science and Engineering, Johns Hopkins University, Baltimore, Maryland, United States of America; 2 Institute for Nanobiotechnology (INBT), Johns Hopkins University, Baltimore, Maryland, United States of America; 3 Department of Physics and Astronomy, Johns Hopkins University, Baltimore, Maryland, United States of America; Ohio State University, UNITED STATES

## Abstract

Tumor vasculature is characterized by a variety of abnormalities including irregular architecture, poor lymphatic drainage, and the upregulation of factors that increase the paracellular permeability. The increased permeability is important in mediating the uptake of an intravenously administered drug in a solid tumor and is known as the enhanced permeation and retention (EPR) effect. Studies in animal models have demonstrated a cut-off size of 500 nm - 1 µm for molecules or nanoparticles to extravasate into a tumor, however, surprisingly little is known about the kinetics of the EPR effect. Here we present a pharmacokinetic model to quantitatively assess the influence of the EPR effect on the uptake of a drug into a solid tumor. We use pharmacokinetic data for Doxil and doxorubicin from human clinical trials to illustrate how the EPR effect influences tumor uptake. This model provides a quantitative framework to guide preclinical trials of new chemotherapies and ultimately to develop design rules that can increase targeting efficiency and decrease unwanted side effects in normal tissue.

## Introduction

In a growing solid tumor, the combination of hypoxic environment and inflammatory response leads to the up-regulation of angiogenic factors and down-regulation of angiogenic inhibitors, promoting the formation of new vessels. This process involves local removal of smooth muscle cells and degradation of basement membrane and extracellular matrix (ECM) by matrix metalloproteinases (MMPs). At the same time, the proliferation of tumor cells causes expansion of the microenvironment and generates local compressive forces [1]. Expansion increases the average spacing between vessels, reducing the supply of nutrients, and creating hypoxic regions in the tumor. The compressive forces generated by tumor growth leads to contraction of blood vessels that contributes to increased resistance to flow. Compressive forces on lymphatic vessels lead to poor lymphatic drainage and increased interstitial fluid pressure.

**Fig 1:**
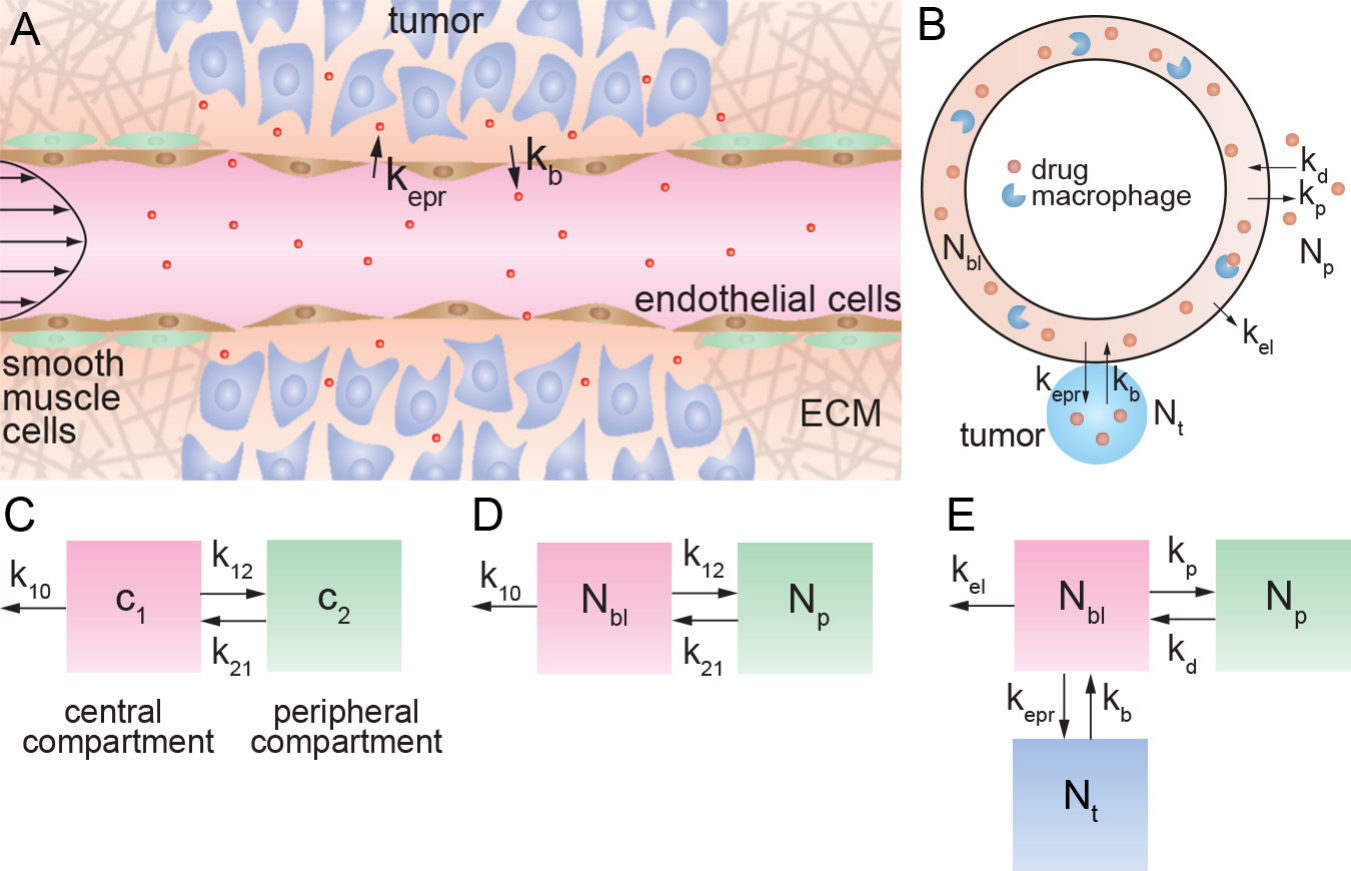
The enhanced permeation and retention (EPR) effect. (A) Schematic illustration of a tumor vessel illustrating loss of smooth muscle cells, local degradation of the extracellular matrix, and increased permeability of the endothelium. (B) Illustration of the pharmacokinetic model taking into account the EPR effect. The rate constants k_p_ and k_d_ describe exchange with the peripheral volume. The rate constants k_epr_ and k_b_ describe extravasation from circulation into the tumor, and intravasation back into the circulation, respectively. The rate constant k_el_ represents clearance by the kidneys, MPS, and any other non-tumor elimination processes, such that when k_b_ = 0, k_10_ = k_epr_ + k_el_ where k_el_ is the elimination rate constant. (C) Standard two compartment model with central and peripheral compartments. c_1_ and c_2_ represent the drug concentration in blood (central compartment) and normal tissue (peripheral compartment), respectively. The first order rate constant k_10_ describes all elimination pathways, including clearance by the kidneys, uptake by the MPS, and tumor accumulation. The first order rate constants k_12_ and k_21_ describe exchange between the two compartments. Note that k_p_ = k_12_, k_d_ = k_21_. (D) Two compartment model defined in terms of the drug amount, where N_bl_ is the amount of drug in blood (mg), and N_p_ is the amount in peripheral tissue (mg). (E) Three compartment model with the addition of a tumor “compartment” where N_t_ is the amount of drug in the tumor. Exchange with the tumor is described by the rate constants k_epr_ and k_b_, respectively. The rate constant k_el_ describes elimination pathways including clearance by the kidneys and uptake by the MPS, but does not include tumor accumulation.

This combination of biochemical and mechanical factors leads to an irregular vascular architecture, increased resistance to blood flow, poor perfusion, and increased permeability. The leakiness of the tumor vasculature is key for systemic delivery of anticancer drugs to a solid tumor, and is known as the Enhanced Permeation and Retention (EPR) effect (Fig 1A) [2, 3]. In animal models, the cut-off size for extravasation from the tumor vasculature varies from 200 nm to 1.2 μm depending on the tumor type [4–6]. A diameter of about 200 nm is often considered an upper limit for successful drug delivery [7, 8].

Despite its critical importance in cancer therapy, surprisingly little is known about the kinetics of the EPR effect. Here we present a model for the pharmacokinetics of a chemotherapeutic drug or nanomedicine that takes into account extravasation from circulation at the tumor site by the EPR effect and intravasation back into circulation. We use data from clinical trials of Doxil and doxorubicin to quantitatively assess the influence of the EPR effect on tumor uptake.

## Model

Accumulation of a drug or nanomedicine in a solid tumor by the EPR effect is dependent on the concentration in blood, and hence requires knowledge of the pharmacokinetics. To evaluate drug accumulation in a solid tumor, we begin with a two compartment model with a central compartment representing the vascular system and highly perfused organs, and a peripheral compartment representing uptake in normal tissue (Fig 1B) [9]. In conventional models, extravasation of a drug at the tumor site is implicitly combined with clearance by the kidneys, mononuclear phagocyte system (MPS), and any other mechanisms, into the rate constant k_10_. To distinguish tumor accumulation by the EPR effect from other elimination pathways, we introduce a tumor “compartment” to the model and define rate constants specifying drug accumulation and removal from the tumor. Drug extravasation into the tumor by the EPR effect is described by the rate constant k_epr_, and intravasation from the tumor back into circulation is described by k_b_. We define the rate constant k_el_ to describe elimination by the kidneys, MPS, and any mechanisms other than tumor uptake. For the case when k_b_ = 0, then k_10_ = k_epr_ + k_el_ (see Text A in S1 File for details). As we show below, the rate of tumor uptake is expected to be slower than the rate of elimination under most clinical conditions and hence k_10_ ≈ k_el_.

In classic pharmacokinetic models, the time dependence of the measured drug concentration in blood or plasma (mg mL^-1^) is fit to a model with first order rate constants describing exchange with the peripheral compartment (k_12_ and k_21_) and elimination (k_10_) (Fig 1C) [9]. A problem in using concentration to describe the amount of drug is that a volume must also be defined. While this is straightforward for the vascular system, it is not well defined for the peripheral tissue or a tumor. To avoid this problem, we define the amount of drug in the different compartments: where N_bl_, N_p_, and N_t_ represent the amount (in mg) of drug in blood, peripheral tissue, and tumor tissue, respectively. To account for the exchange of drug into and out of the peripheral compartment (N_p_), we define k_p_ and k_d_, respectively.

The rate equations can be written in terms of first order rate constants:

$$\frac{dN_{bl}}{dt}=-\left(k_{el}+k_{epr}+k_{p}\right)N_{bl}+k_{d}N_{p}+k_{b}N_{t}$$ 1

$$\frac{dN_{p}}{dt}=k_{p}N_{bl}-k_{d}N_{p}$$ 2

$$\frac{dN_{t}}{dt}=k_{epr}N_{bl}-k_{b}N_{t}$$ 3

Note that this is a dissipated system where mass balance does not hold for these three compartments:

$$\frac{dN_{bl}}{dt}+\frac{dN_{p}}{dt}+\frac{dN_{t}}{dt}=-k_{el}N_{bl}$$ 4

To evaluate the influence of the rate constant for tumor uptake by the EPR effect on tumor accumulation, we consider the pharmacokinetics for Doxil and doxorubicin. Pharmacokinetic data are often empirically fit to an equation consistent with a two compartment model (Text B in S1 File) of the form:

$$N_{bl}=Ae^{-\alpha t}+Be^{-\beta t}$$ 5

where A, B, α, and β are constants. To obtain the rate constants k_12_, k_21_, and k_10_ for Doxil and doxorubicin, we use pharmacokinetic parameters (A, B, α, and β), from a clinical trial (Table 1). The pharmacokinetics are analyzed in terms of the amount of the drug in circulation N_bl_ in units of mg. The rate constants k_12_, k_21_, and k_10_ are related to the parameters A, B, α, and β by the following equations (see Text D in S1 File for derivation):

$$k_{21}=\frac{\beta A+\alpha B}{A+B}$$ 6

$$k_{10}=\frac{\alpha \beta }{k_{21}}$$ 7

$$k_{12}=\alpha +\beta -k_{21}-k_{10}$$ 8

**Table 1:** Pharmacokinetic data for Doxil and doxorubicin obtained from a human clinical trial [12].

	**Doxil**	**Doxorubicin**
**A (mg)**	34.5	28.5
**B (mg)**	61.0	1.1
**α (h** ^-1^ **)**	0.301	11.6
**ß (h** ^-1^ **)**	0.015	0.067
**k**_12_ **, k**_p_ **(h** ^-1^ **)**	0.0956	9.57
**k**_21_ **, k**_d_ **(h** ^-1^ **)**	0.198	0.494
**k**_10_ **(h** ^-1^ **)**	0.0228	1.56

The parameters A, B, α, and β were reported by Gabizon et al. from a clinical trial from a fit to the amount of drug in circulation at time t after administration using a dual exponential decay model: N_bl_(t) = Ae^-αt^ + Be^-βt^, where t is the time and α and β are decay constants. A and B are reported in units of mg, using a blood volume of 5 L. The rate constants k_12_ (= k_p_), k_21_ (= k_d_), and k_10_ are obtained from the parameters A, B, α, and β by assuming a two-compartment model (see Text B in S1 File for details).

Recognizing that k_p_ = k_12_, k_d_ = k_21_, and taking k_el_ = k_10_, the rate equations (Eqs. 1–4) were solved numerically using Matlab (see Text A in S2 File for code) to evaluate the influence of k_epr_ and k_b_ on tumor accumulation.

## Results

### Doxil

Doxil is a liposomal formulation of doxorubicin and was FDA-approved for AIDS-related Kaposi’s sarcoma in 1995, for ovarian cancer in 1999, and for multiple myeloma in 2007 [10]. In 2013, the use of the generic version Lipodox was approved for treatment of ovarian cancer and Kaposi’s sarcoma. Doxil is formulated from a combination of fully hydrogenated soy phosphatidylcholine (HSPC), cholesterol, and a lipid with a polyethylene glycol (PEG) head group (DSPE-PEG2k) [11]. The PEG groups inhibit protein adhesion and slow the rate of uptake by the MPS. Doxil liposomes are about 100 nm in diameter and contain about 10,000–15,000 doxorubcin molecules [11].

The pharmacokinetics of Doxil are characterized by a large area under the curve (AUC), slow clearance rate (CL), small distribution volume (V_d_), and long elimination half time (t_1/2_) [10, 12, 13]. The distribution volume is close to the blood volume and hence the pharmacokinetics for Doxil are sometimes analyzed using a single compartment model. The pegylated lipids in the liposomes result in a long circulation half-time, typically 3–4 days [13].

Pharmacokinetic data were taken from a clinical trial where cancer patients were administered either 25 mg m^-2^ or 50 mg m^-2^ of Doxil, or free doxorubicin [12]. We consider the data from the 50 mg m^-2^ cohort (N = 14 for Doxil and N = 4 for doxorubicin). Gabizon et al. [12] used a two compartment model (Fig 1C) to describe the pharmacokinetic data, and reported the values of A, B, α, and β that best fit the measured blood concentration versus time curves using a biexponential function (Table 1). To convert the dose (which has units of mg m^-2^) and pharmacokinetic constants A and B (which have units of mg L^-1^) to amounts, we use a body surface area of 1.8 m^2^ and a blood volume of 5 L [14].

**Fig 2:**
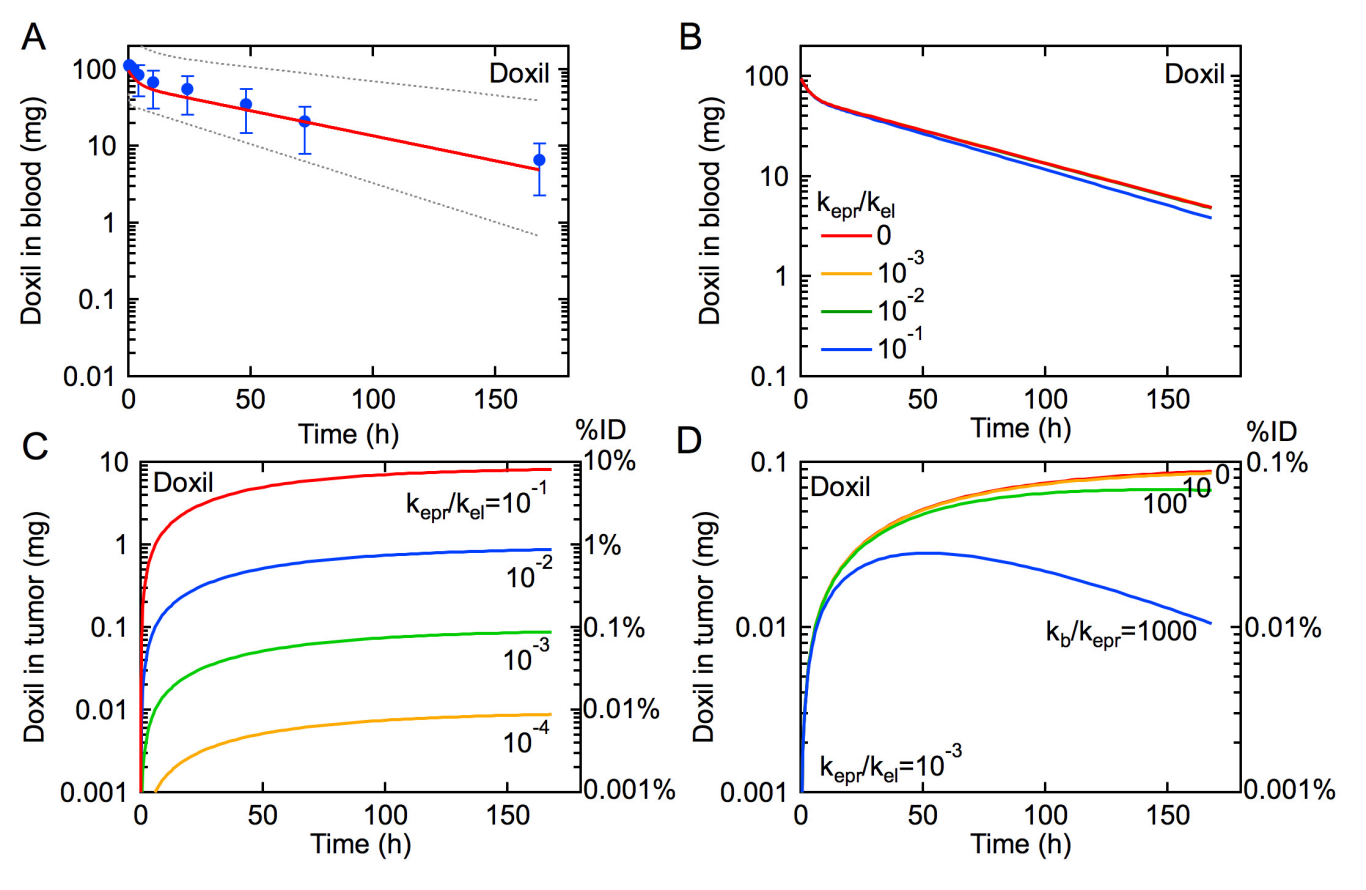
The influence of the EPR effect on the rate of tumor uptake of Doxil for an administered dose of 100 mg (50 mg m^-2^). (A) Pharmacokinetics for Doxil. Symbols are data from a clinical trial reported by Gabizon et al. [12]. The solid red line is obtained from our model using values for k_p_, k_d_, and k_el_ derived from median values of A, B, α, and β reported by Gabizon et al. (Table 1) [12], where k_el_ ~ k_10_ when k_el_ >> k_epr_. The dotted lines represent the pharmacokinetics for the minimum and maximum values of A, B, α, and β. (B) Simulations of the pharmacokinetics for Doxil with k_epr_/k_el_ = 0, 10^–1^, 10^–2^, and 10^–3^, and k_b_ = 0. (C) Amount of Doxil in tumor for k_epr_/k_el_ = 10^–1^, 10^–2^, 10^–3^, and 10^–4^, and k_b_ = 0. (D) Amount of Doxil in tumor for k_epr_/k_el_ = 10^–3^ and k_b_/k_epr_ = 0, 10, 100, 1000. The amount of Doxil in tumor represents the total amount of doxorubicin.

Values for the rate constants k_12_, k_21_, and k_el_ for Doxil were obtained from the values for A, B, α, and β (Table 1) from the clinical trial using a standard two compartment model (Fig 1C) [12]. In our model, the amount of drug is specified for each compartment instead of concentration (Fig 1D). We take k_p_ = k_12_, k_d_ = k_21_, and k_el_ ~ k_10_ and apply these rate constants to our three compartment model while initially assuming a negligible rate of extravasation from the EPR effect (k_epr_ ~ 0) (Fig 1E). As anticipated, the numerical solution of the rate equations shows excellent agreement with the pharmacokinetic data (Fig 2A), since we are simulating a two compartment model when k_epr_ = 0. Next, we introduce the tumor compartment and increase values of k_epr_, while keeping k_p_, k_d_, and k_el_ fixed, to determine detectable changes in the pharmacokinetics due to the EPR effect.

We calculate the pharmacokinetics for Doxil using the values for k_p_, k_d_, and k_el_ obtained from Gabizon et al. (Table 1) and different ratios of k_epr_/k_el_ (10^–1^, 10^–2^, 10^–3^, and 0) with k_b_ fixed at 0 (Fig 2B). By exploring a range of ratios of k_epr_/k_el_, we can simulate the EPR effect for a variety of tumors with varying degrees of drug absorption relative to other elimination pathways. When the rate constant for tumor uptake by the EPR effect is smaller than the rate constant for elimination (k_epr_/k_el_ < 0.1), the influence of the EPR effect is not seen in the pharmacokinetics. Therefore, as long as k_el_ >> k_epr_, the pharmacokinetics for subjects with and without tumors would be indistinguishable (Fig 2B). As we show below, k_epr_ is expected to be much slower than k_el_ for most cases of clinical interest.

From the numerical simulations we can extract the amount of Doxil accumulated in the tumor (N_t_) for the different values of k_epr_ (Fig 2C). For k_epr_/k_el_ = 10^–1^, tumor uptake by the EPR effect increases quickly in the first few hours and then asymptotically approaches a maximum corresponding to about 10% of the initial dose after 4 days. Decreasing the value of k_epr_/k_el_ results in slower accumulation and a maximum that decreases by an order of magnitude for every order of magnitude decrease in k_epr_/k_el_.

In xenograft mouse models, the maximum reported accumulation of nanomedicines is around 10% of the initial dose [15]. However, tumor xenografts are usually formed from cell lines that result in highly vascularized and leaky tumors, and hence the fraction of the initial dose accumulated in the tumor and k_epr_ is expected to be much higher than in a human subject. Even when using overestimated values of k_epr_ to achieve 10% ID accumulated in tumors (e.g. k_epr_/k_el_ = 10^–1^), there is only a negligible change in the pharmacokinetics compared to when k_epr_ = 0 (Fig 2B–2C). This result implies that tumor accumulation by the EPR effect is not a significant sink for the drug in circulation, i.e k_el_N_bl_ >> k_epr_N_bl_.

So far we have assumed that there is no intravasation back into circulation (k_b_ = 0), corresponding to the case where all extravasated drug remains in the tumor. This could occur by rapid uptake and sequestration of the drug by tumor cells close to the vessels or by active targeting resulting in irreversible binding to tumor cells. We next fix k_epr_/k_el_ = 10^–3^, and assess how k_b_ influences drug accumulation at the tumor site.

When k_b_ ≈ k_epr_ there is relatively little difference in tumor accumulation (Fig 2D). However, for larger values of k_b_ (k_b_/k_epr_ > 10), the amount of drug in the tumor reaches a maximum and then decreases at longer times. With increasing k_b_, the maximum occurs at shorter times and the rate of intravasation from the tumor increases. This effect is due to the fact that initially the amount of drug in the tumor is small, and hence the rate of transport from the tumor back to the vascular system (k_b_N_t_) is also small. At longer times the amount of drug in circulation decreases resulting in a decrease in the rate of extravasation to the tumor, and the amount of drug in the tumor increases, resulting in an increase in the rate of intravasation. Eventually, the rate of intravasation becomes larger than the rate of extravasation. A similar maximum in tumor accumulation has been reported for administration of a pegylated liposome in a mouse model [16]. These simulations highlight an important insight that is often neglected: the EPR effect allows transport in both directions—into and out of the tumor.

The model presented here provides a framework to assess the influence of the EPR effect on tumor uptake. The rate of uptake is described by k_epr_N_bl_ highlighting the fact that the rate constant represents extravasation of the drug along the total length of the tumor vasculature. Since it is difficult to measure the length of tumor vasculature, normalization by the tumor size may be a reasonable alternative. Since the rate constant for tumor uptake represents the leakiness of the tumor vasculature, it is expected to be dependent on the tumor type and is likely to vary from patient to patient. This is discussed in more detail below.

The rate of intravasation from the tumor back to the vasculature is described by k_b_N_t_. For a drug molecule in circulation under pulsatile flow, the residence time of a drug molecule in the vicinity of a defect in the endothelium of the tumor vasculature is expected to be relatively short, and hence the probability of extravasation is expected to be low. Conversely, the residence time for a drug molecule in the vicinity of a defect on the tumor side of the endothelium is expected to be much higher since the rate of interstitial flow is much lower than blood flow. Consequently, the probability of extravasation is expected to be lower than the probability of intravasation (i.e. k_b_ > k_epr_). While other processes such as diffusive transport and cellular uptake are not explicitly determined in this model (Fig 1), their effects on the rate of intravasation are included in k_b_. For example, in tumors where diffusive transport is fast or where active targeting limits intravasation, the rate of intravasation (k_b_N_t_) is expected to be much smaller than the rate of extravasation (k_epr_N_bl_).

In this model we make no assumptions about the fate of the drug after extravasation. Diffusive transport into the tumor, non-specific binding or targeting to cell membranes, cellular uptake, and metabolism will all influence the amount of free drug in the tumor close to the vasculature that is available for intravasation back into circulation. With experimental progress in quantitative assessment of the EPR effect, future models can incorporate these downstream processes.

### Doxorubicin

To provide a comparison to tumor accumulation for Doxil, we also assess the influence of the EPR effect on tumor accumulation of doxorubicin. Doxorubicin is an anthracyline commonly used in the treatment of a wide range of cancers. The distribution volume for free doxorubicin is very large illustrating that a significant amount of the drug is taken up in normal tissues [17–20]. The AUC for doxorubicin is about three orders of magnitude smaller than Doxil resulting in a clearance rate about three orders of magnitude larger [17–20]. The elimination half-time for doxorubicin is about 20–25 h.

**Fig 3:**
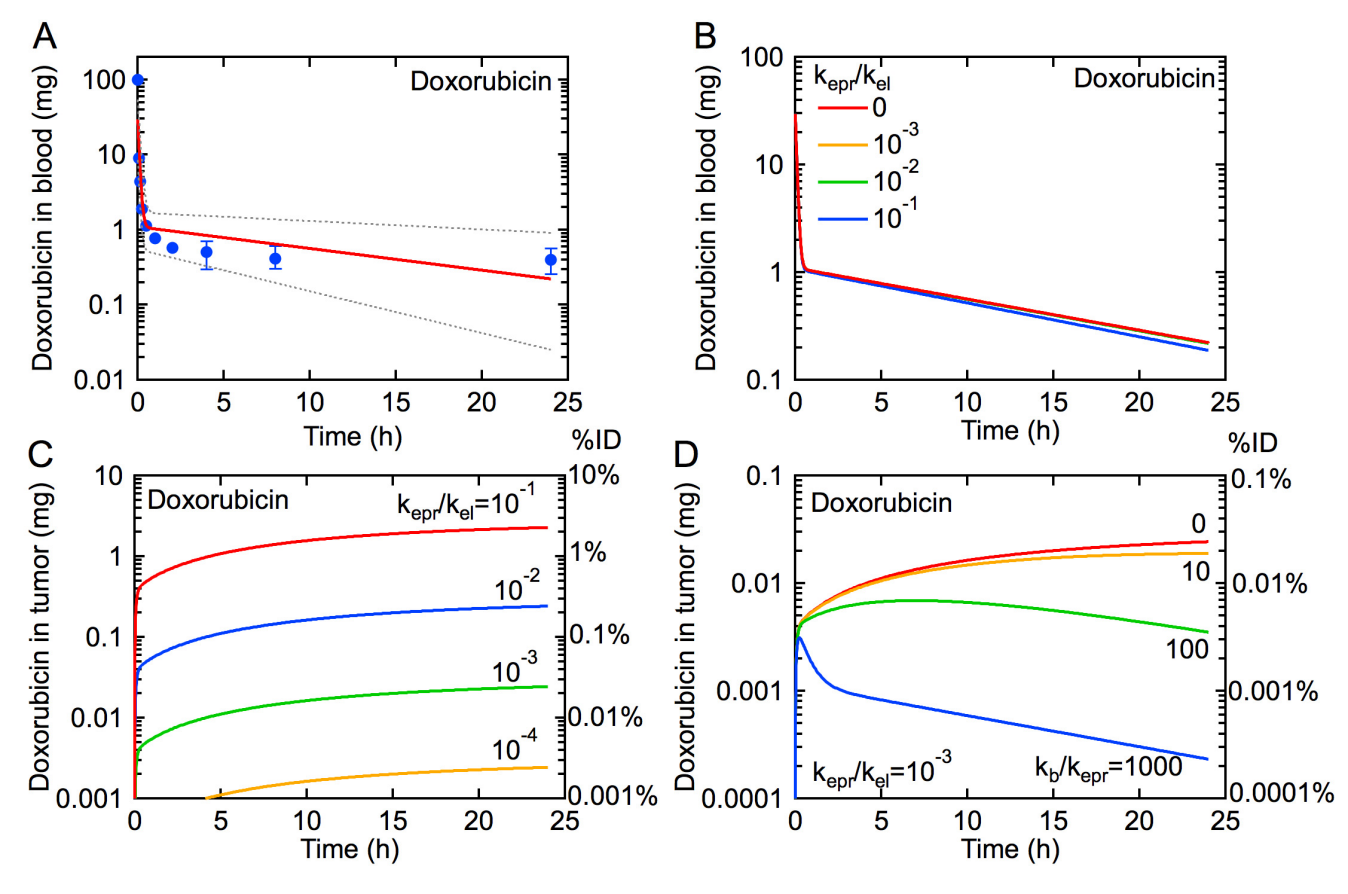
The influence of the EPR effect on the rate of tumor uptake of doxorubicin for an administered dose of 100 mg (50 mg m^-2^). (A) Pharmacokinetics for doxorubicin. Symbols are data from a clinical trial reported by Gabizon et al. [12]. The solid red line is obtained from our model using values for k_p_, k_d_, and k_el_ derived from median values of A, B, α, and β reported by Gabizon et al. [12] (Table 1), where k_el_ ~ k_10_ when k_el_ >> k_epr_. The dotted lines represents the pharmacokinetics for the minimum and maximum values of A, B, α, and β. (B) Simulations of the pharmacokinetics for doxorubicin with k_epr_/k_el_ = 0, 10^–1^, 10^–2^, and 10^–3^, and k_b_ = 0. (C) Amount of doxorubicin in tumor for k_epr_/k_el_ = 10^–1^, 10^–2^, 10^–3^, and 10^–4^, and k_b_ = 0. (D) Amount of doxorubicin in tumor for k_epr_/k_el_ = 10^–3^ and k_b_/k_epr_ = 0, 10, 100, 1000.

Pharmacokinetic data for doxorubicin were obtained from Gabizon et al. (Fig 3A) [12]. Similar values have been reported in other clinical trials [17–19]. Values for the pharmacokinetic parameters A, B, α, and β, as well as the rate constants k_12_, k_21_, and k_10_, are provided in Table 1.

As described previously for Doxil, tumor uptake of doxorubicin by the EPR effect is not expected to be seen in the pharmacokinetics due to the dominant contribution of non-tumor elimination pathways (Fig 3B). First we consider tumor accumulation for different values of k_epr_/k_el_ with k_b_ = 0 (Fig 3C). The increase in tumor accumulation is rapid during the initial distribution phase. However, as the amount in blood decreases rapidly in the first few minutes, tumor accumulation slows significantly during the elimination phase. The maximum amount of drug accumulated in the tumor is lower than for Doxil due to the lower concentration in blood at all times and the faster clearance (k_el_) (see Table 1). Decreasing the value of k_epr_/k_el_ results in significantly lower accumulation in the tumor.

Fixing k_epr_/k_el_ = 10^–3^, we assess the influence of intravasation (k_b_) on tumor accumulation (Fig 3D). For k_b_/k_epr_ ≥ 10, tumor accumulation reaches a maximum and subsequently decreases. The rate of decrease is much faster than for Doxil, especially for larger values of k_b_/k_epr_ as the amount of drug in circulation decreases rapidly in the distribution phase.

### Drug delivery and distribution within the tumor microenvironment

In Figs 2 and 3 we show the global accumulation of anticancer drugs within a tumor for an initial dose of 100 mg. To illustrate drug accumulation in the local tumor microenvironment, we consider a 100 μm length of tumor vessel. Assuming a tumor volume of 1 cm^3^ and vessel length density of 150 mm mm^-3^, the total length of tumor vasculature is about 150 m with an average vessel diameter of 30 μm (S1 Table) [21]. A 100 μm segment of a 30 μm diameter vessel has about 3–4 endothelial cells around the circumference and is about 3–4 cell lengths long.

**Fig 4:**
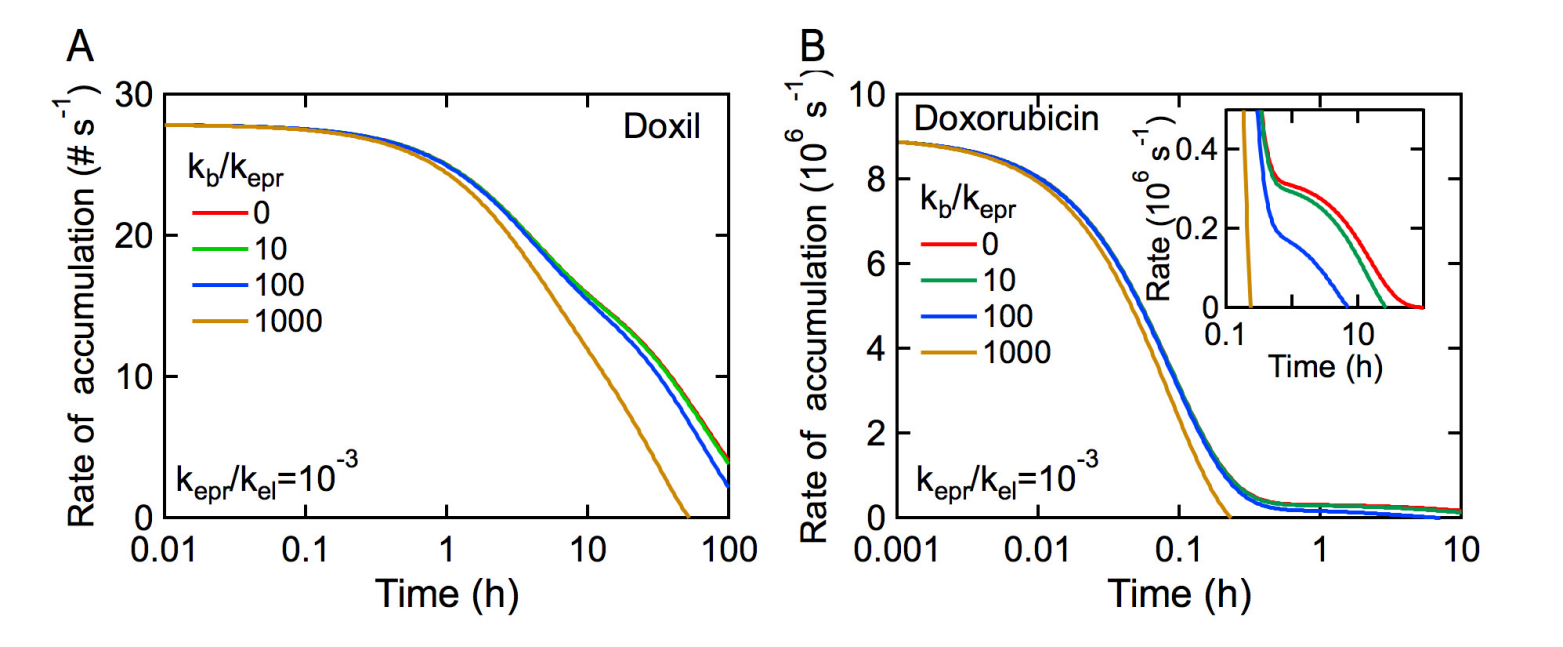
Drug accumulation rate in the tumor per 100 μm vessel length assuming a 1 cm^3^ tumor with 150 m of vessels (150 mm mm^-3^). (A) Doxil and (B) Doxorubicin. Values for k_p_, k_d_, and k_el_ are given in Table 1, where k_el_ ~ k_10_ when k_el_ >> k_epr_. In both cases k_epr_/k_el_ = 10^–3^.

Taking k_epr_/k_el_ = 10^–3^ (0.1% ID with k_b_ = 0), the local accumulation of Doxil along a 100 μm segment corresponds to 10^4^ to 10^6^ liposomes, depending on the value of k_b_. For doxorubicin, the total accumulation along a 100 μm vessel segment is 10^7^–10^10^ molecules. The derivative of tumor accumulation versus time is the rate of extravasation. Based on the pharmacokinetics of Doxil and taking k_epr_/k_el_ = 10^–3^, the accumulation rate for a 100 μm vessel segment is about 28 liposomes per second for the first hour after administration, independent of the magnitude of the back rate constant k_b_ (Fig 4A). The accumulation rate remains relatively high, greater than 10 liposomes per second, for a day or more depending on the value of constant k_b_. The high accumulation rate is due to the long circulation time of the liposomes which also minimizes the effect of intravasation back into circulation except at very large values of k_b_.

In contrast, the rate of tumor accumulation of doxorubicin decreases rapidly from about 9 x 10^6^ molecules per second to less than about 1 x 10^6^ per second within 10 minutes (Fig 4B). This is due to the rapid uptake in normal tissue, which quickly reduces the concentration in circulation. If the rate constant for intravasation is small, then the accumulation rate in the tumor is about 0.4 x 10^6^ molecules per second, a factor of 20 lower than the initial accumulation rate, up to about 10 hours. For both Doxil and doxorubicin, if the rate constant for intravasation is large, typically k_b_/k_epr_ > 100, the accumulation rate eventually becomes negative indicating a net loss from the tumor. For a drug where the blood concentration remains high, the influence of k_b_ on the accumulation rate is delayed until relatively long times after administration. However, for drugs with rapid absorption in normal tissue (i.e. large distribution volume), the intravasation rate constant k_b_ can significantly reduce tumor accumulation.

## Discussion

### Implications of the model

The EPR effect plays an important role in modulating uptake of a drug to a solid tumor, and yet surprisingly little is known about the kinetics of tumor uptake. Developing a quantitative framework to describe the EPR effect is crucial to guide the design of drug delivery systems and ultimately in providing input in dosing and clinical management. Here we present a model to provide quantitative analysis of the EPR effect that uses pharmacokinetic measurements of the time dependent blood concentration as an input parameter. The rate constants k_epr_ and k_b_ describe extravasation to the tumor site and intravasation back into circulation, respectively. The amount of drug extravasating from the vascular system at a tumor site is dependent on the competition between the EPR effect and elimination due to the kidneys, MPS, and other non-tumor pathways. For drugs such as Doxil with a long elimination half-time, tumor accumulation increases rapidly. In contrast, tumor accumulation is significantly lower for drugs with very short elimination half-times, such as Doxorubicin, for the same value of k_epr_. Intravasation from the tumor site back into circulation is an important and often ignored factor in determining tumor accumulation. As the rate constant for intravasation k_b_ increases, loss of drug from the tumor to circulation becomes significant.

The model presented here provides quantitative insight into the EPR effect and the coupling between pharmacokinetics and tumor accumulation that will be useful for basic science and preclinical trials of new drugs and nanomedicines. Preclinical trials that quantify both pharmacokinetics and tumor accumulation (% ID) can be used to predict k_epr_ values (Figs 2C and 3C). For example, we limited k_epr_/k_el_ to achieve tumor accumulations that are consistent with those reported *in vivo* in a mouse model, ranging from 0.01–10% and 0.001–1% ID for Doxil and doxorubicin respectively [15, 22, 23]; free doxorubicin reaches maximum accumulation faster in tumors but remains at concentrations roughly 10 times less than liposomal formulations due to differences in pharmacokinetics and uptake mechanisms [12, 23, 24]. Furthermore, this model can be used to predict how changes in nanomedicine design that modulate pharmacokinetics will influence tumor accumulation by modulating k_el_, k_epr_, and k_b_.

This model also raises a number of key scientific questions concerning the rate constants k_epr_ and k_b_. Importantly, the model provides a quantitative framework to discuss these questions. (1) What is the spatial variability in tumor leakiness? (2) What is the variability in leakiness from patient-to-patient within a tumor type? (3) What is the variability in leakiness across different tumor types?

There are two sources of spatial variability in tumor leakiness and tumor uptake: (1) intrinsic variations in leakiness per unit length of vessel, and (2) and regional variations in vascular density. Non-uniform extravasation and discontinuous pooling of pegylated liposomes in a xenograft mouse model [6, 25] suggest spatial variations in k_epr_. Vascular leakiness is expected to be greater at angiogenic sprouts compared to developed neovasculature. The accumulation of radiolabeled stealth liposomes was found to be about 10 times greater in the periphery compared to the necrotic core 24 hours after administration in a mouse tumor xenograft [26]; this is likely due to a higher vascular density at the tumor periphery and the increased interstitial pressure at the tumor core [27–29]. These results suggest that spatial variations in accumulation of an order of magnitude are reflected in k_epr_ (Fig 2C). While in our model, there is a single tumor compartment and rate constant for extravasation, k_epr_ (Fig 1E), additional tumor compartments and rate constants accounting for spatial variation (e.g. tumor core, periphery, interstitial pressure) may be introduced. Knowledge of the variation of k_epr_ within a tumor may be used to determine a range of doses that distributes a therapeutically effective concentration of drug to all regions of the tumor.

The rate constants k_epr_ and k_b_ have units of s^-1^ and are dependent on the length and leakiness of the tumor vasculature and hence are expected to be dependent on tumor type and exhibit patient-to-patient variations. Normalizing the rate constants to unit volume would allow comparison of different tumor sizes. Tumor accumulation of radiolabeled liposomes in a clinical study of patients with various tumor types ranged from 0.3 to 3.6% of the initial dose (ID) 72 hours post injection. When normalized by tumor weight (% ID/kg), uptake into breast tumors was roughly 3 times lower than lung tumors and 6 times lower than squamous cell cancer of the head and neck [22]. These patient-to-patient variations may be attributed to differences in density, structure, and inflammation of the tumor vasculature affecting blood flow and drug accumulation. The quantification of both drug accumulation and tumor perfusion will improve our understanding of the influence of tumor blood flow and vascularity on the EPR effect.

Measurement of the rate constants for extravasation and transport back into circulation (k_epr_ and k_b_) for different tumor types and assessment of patient-to-patient variations may improve dosing and clinical management. This requires detailed time-dependent measurements of both the pharmacokinetics and tumor accumulation, but these data do not exist for human clinical trials and are without sufficient granularity from preclinical studies in animal models. However, *in vivo* techniques, such as PET imaging of tumor accumulation of a labeled surrogate (e.g. radiolabeled pegylated liposomes) [22], may provide the temporal resolution needed to determine k_epr_ and k_b_ for individual patients. Similarly, these rate constants can be extracted from preclinical trials in animal models that measure drug accumulation as % ID in tumor homogenates at frequent time points. The ratio of k_b_/k_epr_ can provide a prognostic value of tumor response, since it captures both the rate of accumulation and clearance of the drug, which have been correlated with tumor regression [27]. Therapeutic efficacy and dosing intervals can be predicted and optimized from these rates constants.

## Conclusions

This model represents a first step in quantitative analysis of tumor accumulation of a drug or nanomedicine by the EPR effect. There are models for almost every other step in drug delivery for cancer therapy (e.g. pharmacokinetics, diffusion, uptake, tumor growth, survival rates, etc.), but not the crucial EPR effect. Using pharmacokinetic data for Doxil and doxorubicin we show how the EPR effect influences tumor accumulation for different values of the rate constants for tumor uptake by the EPR effect and intravasation back into circulation. Modeling results show the kinetics of drug accumulation with time. By taking into account intravasation back into circulation, tumor uptake shows a characteristic maximum, with loss of drug from the tumor at longer times. This model provides a quantitative framework to guide preclinical trials of new chemotherapeutic delivery vehicles and ultimately to develop design rules that can increase targeting efficiency and decrease unwanted side effects in normal tissue.

## Supporting Information

S1 File Supplementary information. (1) Pharmacokinetic Model. (2) Relationship between k_10_ and k_el_. (3) Obtaining rate constants k_10_, k_12_, k_21_ from pharmacokinetic data for Doxil and doxorubicin. (4) Simulations. (5) Derivation of the relationship between the pharmacokinetic parameters A, B, α, and β and the rate constants k_10_, k_12_, and k_21_.
(DOCX)

S2 File Matlab simulation code. (DOCX)

S1 Table The architecture of tumor vasculature. (DOCX)
