# Supplementary material for: Quantitative Analysis of the Enhanced Permeation and Retention (EPR) Effect
Source: PLoS One. 2015 May 4;10(5):e0123461. doi: 10.1371/journal.pone.0123461 (PMC4418820; doi:10.1371/journal.pone.0123461)
Supplement: S1 File — (1) Pharmacokinetic Model. (2) Relationship between k10 and kel. (3) Obtaining rate constants k10, k12, k21 from pharmacokinetic data for Doxil and doxorubicin. (4) Simulations. (5) Derivation of the relationship between the pharmacokinetic parameters A, B, α, and β and the rate constants k10, k12, and k21. (DOCX) [file pone.0123461.s001.docx]

# Quantitative Analysis of the Enhanced Permeation and Retention (EPR) Effect

Andrew D. Wong,^1,2¶^ Mao Ye,^3¶^ Martin B. Ulmschneider,^1,2^ and Peter C. Searson^1,2,3^*

^1^ Department of Materials Science and Engineering, Johns Hopkins University, Baltimore, Maryland, United States of America

^2^ Institute for Nanobiotechnology (INBT), Johns Hopkins University, Baltimore, Maryland, United States of America

^3^ Department of Physics and Astronomy, Johns Hopkins University, Baltimore, Maryland, United States of America

* Corresponding author

E-mail: searson@jhu.edu

^¶^ These authors contributed equally to this work.

**Supplementary Information**

# Fig. A in S1 File. Pharmacokinetic Model

# Text A in S1 File. Relationship between k_10_ and k_el_

# Text B in S1 File. Obtaining rate constants k_10_, k_12_, k_21_ from pharmacokinetic data for Doxil and doxorubicin

# Text C in S1 File. Simulations

# Text D in S1 File. Derivation of the relationship between the pharmacokinetic parameters A, B, α, and β and the rate constants k_10_, k_12_, and k_21_.

# Figure A. Pharmacokinetic Model


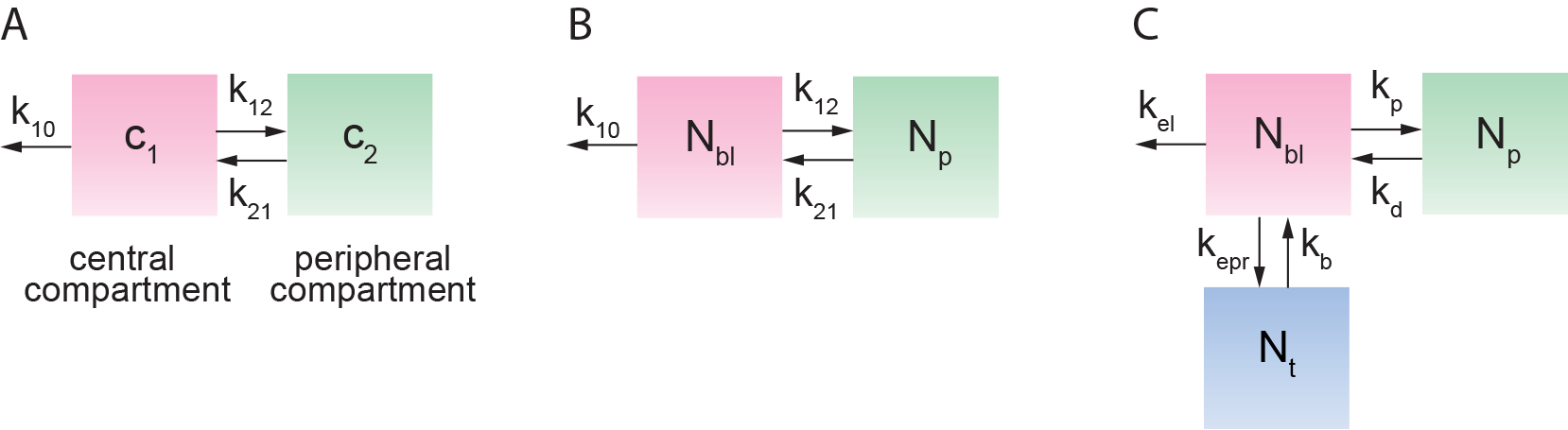


**Figure A**. (A) Standard two compartment model with central and peripheral compartments. c_1_ and c_2_ represent the drug concentration in blood (central compartment) and normal tissue (peripheral compartment), respectively. The first order rate constant k_10_ describes all elimination pathways, including clearance by the kidneys, uptake by the MPS, and tumor accumulation. The first order rate constants k_12_ and k_21_ describe exchange between the two compartments. (B) Two compartment model defined in terms of the drug amount, where N_bl_ is the amount of drug in blood (mg), and N_p_ is the amount in peripheral tissue (mg). (C) Three compartment model with the addition of a tumor “compartment” where N_t_ is the amount of drug in the tumor. Exchange with the tumor is described by the rate constants k_epr_ and k_b_, respectively. The rate constant k_el_ describes elimination pathways including clearance by the kidneys and uptake by the MPS, but does not include tumor accumulation. Note that k_p_ = k_12_, k_d_ = k_21_.

# Text A. Relationship between k_10_, k_el_, and k_epr_

Comparison of the rates of elimination in the standard two compartment model and the three compartment model with a tumor compartment (Fig. A in S1 File) gives:

$-k_{10}N_{bl}=-\left(k_{el}+k_{epr}\right)N_{bl}+k_{b}N_{t}$ (2.1)

Rearranging:

$k_{10}=k_{el}+k_{epr}+k_{b}\frac{N_{t}}{N_{bl}}$ (2.2)

When k_b_ = 0 or as long as k_b_N_t_/N_bl_ << k_el_ and k_epr_, then

$k_{10}\approx k_{el}+k_{epr}$ (2.3)

Since k_el_ is expected to be larger than k_epr_ for most cases of clinical interest, k_10_ ≈ k_el_.

# Text B. Obtaining rate constants k_10_, k_12_, k_21_ from pharmacokinetic data for Doxil and doxorubicin

Pharmacokinetic data are often empirically fit to an equation consistent with a two compartment model (Fig. A in S1 File) of the form:

$N_{bl}=Ae^{-\alpha t}+Be^{-\beta t}$ (3.1)

where A, B, α, and β are constants. To obtain the rate constants k_10_, k_12_, and k_21_ for Doxil and doxorubicin, we use pharmacokinetic parameters (A, B, α, and β), reported by Gabizon et al.[[1](#_ENREF_1)] from a cohort of patients from a clinical trial who were administered a dose of 50 mg m^-2^.

The pharmacokinetics are analyzed in terms of the amount of the drug in circulation N_bl_ in units of mg. It is evident that c_bl_ = N_bl_/V_bl_ and we take V_bl_ = 5 L. The units of α and β are h^-1^ independent of whether the pharmacokinetics are described in terms of the drug amount or concentration. The units of A and B are mg as opposed to mg L^-1^ if the pharmacokinetic data are in units of concentration (mg L^-1^).

The rate constants k_10_, k_12_, and k_21_ are related to the parameters A, B, α, and β by the following equations:[[2](#_ENREF_2)]

$k_{21}=\frac{\beta A+\alpha B}{A+B}$ (3.2)

$k_{10}=\frac{\alpha\beta}{k_{21}}$ (3.3)$k_{12}=\alpha+\beta-k_{21}-k_{10}$ (3.4)

Note that:

$A+B=N_{bl}(0)=N_{0}$ (3.5)

where N_0_ is the initial dose in mg. The derivation of these equations is provided below.

1. Gabizon, A.; Catane, R.; Uziely, B.; Kaufman, B.; Safra, T.; Cohen, R.; Martin, F.; Huang, A.; Barenholz, Y. Prolonged circulation time and enhanced accumulation in malignant exudates of doxorubicin encapsulated in polyethylene-glycol coated liposomes. *Cancer Res.* 1994, 54, 987-992.

2. Gibaldi, M.; Perrier, D., *Pharmacokinetics*. 2nd ed., (Dekker, New York, 1999).

# Text C. Simulations

Our model (Fig. A in S1 File) is described by the following first-order differential rate equations:

$\frac{dN_{bl}}{dt}=-\left(k_{el}+k_{epr}+k_{p}\right)N_{bl}+k_{d}N_{p}+k_{b}N_{t}$ (4.1)

$\frac{dN_{p}}{dt}=k_{p}N_{bl}-k_{d}N_{p}$ (4.2)

$\frac{dN_{t}}{dt}=k_{epr}N_{bl}-k_{b}N_{t}$ (4.3)

$\frac{dN_{bl}}{dt}+\frac{dN_{p}}{dt}+\frac{dN_{t}}{dt}=-k_{epr}N_{bl}$ (4.4)

After obtaining the rate constants k_p_ (= k_12_) and k_d_ (= k_21_) and k_10_ from pharmacokinetic parameters (A, B, α, and β), and taking k_el_ = k_10_, the rate equations were solved numerically using Matlab (see Text A in S2 File for code) to evaluate the influence of k_epr_ and k_b_ on tumor accumulation.

# Text D. Derivation of the relationship between the pharmacokinetic parameters A, B, α, and β and the rate constants k_12_, k_21_, and k_10_.

The differential equations describing the drug amount in the central compartment (N_bl_, mg) and peripheral compartment (N_p_, mg) (see Fig. A in S1 File) are given by:

$\frac{dN_{bl}}{dt}=-(k_{10}+k_{12})N_{bl}+k_{21}N_{p}$ (6.1)

$\frac{dN_{p}}{dt}=k_{12}N_{bl}-k_{21}N_{p}$ (6.2)

where k_10_ (h^-1^) is the first-order elimination rate constant, k_12_ (h^-1^) is the first-order rate constant for drug transfer from central to the peripheral compartment, and k_21_ (h^-1^) is the first-order rate constant for drug transfer from the peripheral to central compartment.

Equations 6.1 and 6.2 can be solved in the following way. Rearranging equation 6.2 we obtain:

$N_{p}=\frac{1}{k_{21}}\left(\frac{dN_{bl}}{dt}+(k_{12}+k_{10})N_{bl}\right)$ (6.3)

Differentiating equation 6.3 we obtain:

$\frac{dN_{p}}{dt}=\frac{1}{k_{21}}\left(\frac{d^{2}N_{bl}}{dt^{2}}+(k_{12}+k_{10})\frac{dN_{bl}}{dt}\right)$ (6.4)

Substituting equation 6.4 into equation 6.2 we eliminate N_p_ and obtain an expression in terms of N_bl_:

$\frac{d^{2}N_{bl}}{dt^{2}}+(k_{12}+k_{10}+k_{21})\frac{dN_{bl}}{dt}+k_{10}k_{21}N_{bl}=0$ (6.5)

The general solution to equation 6.5 is of the form N_bl_ = e^-λt^, from which we obtain:

$\lambda^{2}-(k_{12}+k_{10}+k_{21})\lambda+k_{10}k_{21}=0$ (6.6)

The coefficients (α and β) of this quadratic equation:

$\alpha=\frac{k_{12}+k_{21}+k_{10}+\sqrt{(k_{12}+k_{21}+k_{10})^{2}-4k_{21}k_{10}}}{2}$ (6.7)

$\beta=\frac{k_{12}+k_{21}+k_{10}-\sqrt{(k_{12}+k_{21}+k_{10})^{2}-4k_{21}k_{10}}}{2}$ (6.8)

The solution to equation 6.5 is a linear combination of the general solution:

$N_{bl}=Ae^{-\alpha t}+Be^{-\beta t}$ (6.9)

The values of A and B are obtained from the boundary conditions. Substituting the boundary condition that N_bl_ = N_0_ at t = 0 into equation 6.9 we obtain:

$A+B=N_{0}$ (6.10)

Substituting the boundary condition that N_p_ = 0 at t = 0 into equation 6.1:

$\frac{dN_{bl}}{dt}=-(k_{10}+k_{12})N_{bl}$ (6.11)

Differentiating equation 6.9 we obtain:

$\frac{dN_{bl}}{dt}=-\alpha Ae^{-\alpha t}-\beta Be^{-\beta t}$ (6.12)

Recalling that N_bl_ = N_0_ at t = 0, we obtain:

$\alpha A+\beta B=(k_{10}+k_{12})N_{0}$ (6.13)

Substituting equation 6.10 into 6.13, we obtain expressions for the coefficients A and B:

$A=\frac{k_{12}+k_{10}-\beta}{\alpha-\beta}N_{0}$ (6.14)

$B=\frac{k_{12}+k_{10}-\alpha}{\beta-\alpha}N_{0}$ (6.15)

Equations 6.7, 6.8, 6.14, and 6.15 define the pharmacokinetic parameters A, B, α, and β in terms of the rate constants k_12_, k_21_, k_10_. These rate constants can be used to determine the time dependent amount in circulation (equation 6.9).

From equation 6.3 we can calculate the time dependent amount in the periphery:

$N_{p}=\frac{1}{k_{21}}\left(\frac{dN_{bl}}{dt}+(k_{12}+k_{10})N_{bl}\right)=Ae^{-\alpha t}\left(\frac{k_{12}+k_{10}-\alpha}{k_{21}}\right)+Be^{-\beta t}\left(\frac{k_{12}+k_{10}-\beta}{k_{21}}\right)$ (6.16)
