# Supplementary material for: Quantitative Analysis of the Enhanced Permeation and Retention (EPR) Effect
Source: PLoS One. 2015 May 4;10(5):e0123461. doi: 10.1371/journal.pone.0123461 (PMC4418820; doi:10.1371/journal.pone.0123461)
Supplement: S2 File — (DOCX) [file pone.0123461.s002.docx]

# Text A in S2 File: Simulation code

% EPR Model

% Mao Ye, Peter C. Searson

% Johns Hopkins University

% EPR takes pharmacokinetic parameters and calculates tumor

% accumulation based on user-defined values of the rate constants

% associated with the EPR effect.

% Sample input

% (a) A = 6.9, B = 12.2, alpha = 0.3014, beta = 0.015

% (b) N0 = 19.1, kel = 0.0228, kp = 0.0956, kd = 0.198

% kepr = 0.000228, kb = 2.23, T = 168, filename = 'epr'

function EPR()

fprintf('***************************************************************\n');

fprintf('Welcome to the EPR simulation program\n');

fprintf('This simulations determines the tumor accumulation');

fprintf('of an anti-cancer drug or nanomedicine.\n');

fprintf('You can choose to implement a one compartment model \n');

fprintf('or a two compartment model with your own');

fprintf('pharmacokinetic parameters.\n');

fprintf('***************************************************************\n\n');

%% Specify name of data folder

ResFolder = 'result';

if (~exist(ResFolder,'dir'))

mkdir(ResFolder);

end

%% Step 1: enter parameters based on pharmacokinetic parameters as (a)A, B, alpha, beta; or (b)N0, kel, kp, kd

fprintf('---Step 1: enter parameters related to dose and rate constants---\n');

s1 = 'o'; % s1 is a string to control the input/output of step 1

while(s1 ~= 'a' && s1 ~= 'b')

fprintf('---------------------------------------------------------\n');

fprintf('(a): A(mg), B(mg), alpha(h^-1), beta(h^-1) \n');

fprintf('(b): N0(mg), kel (h^-1), kp(h^-1), kd(h^-1) \n');

s1 = input('Please select one combination of the above parameters to enter (a or b): ', 's');

if(s1 == 'a') % enter parameters (a)

fprintf('You picked (a): A(mg), B(mg), alpha(h^-1), beta(h^-1) \n');

% A, B, alpha, beta are pharmacokinetic parameters

A = input('A(mg) = ');

B = input('B(mg) = ');

alpha = input('alpha(h^-1) = ');

beta = input('beta(h^-1) = ');

fprintf('You entered: A(mg)=%d, B(mg)=%d, alpha(h^-1)=%d, beta(h^-1)=%d\n', A,B,alpha,beta);

% calculate N0, kel, kp, kd based on A, B, alpha, beta

N0 = A+B;

kd = (A*beta+B*alpha)/(A+B);

kel = alpha*beta/kd;

kp = alpha+beta-kd-kel;

fprintf('---------------------------------------------------------\n');

fprintf('Calculated: N0(mg)=%d, kel(h^-1)=%d, kp(h^-1)=%d, kd(h^-1)=%d\n', N0, kel, kp, kd);

break;

elseif (s1 == 'b')

fprintf('You picked (b): N0(mg), kel(h^-1), kp(h^-1), kd(h^-1) \n');

% N0 is initial dose

% if kel, kp, kd are known, input them directly

N0 = input('N0(mg) = ');

kel = input('kel(h^-1) = ');

kp = input('kp(h^-1) = ');

kd = input('kd(h^-1) = ');

fprintf('You entered: N0(mg)=%d, kel(h^-1)=%d, kp(h^-1)=%d, kd(h^-1)=%d\n', N0, kel,kp,kd);

tmp1 = kp+kd+kel;

tmp2 = kd*kel;

% calculate A, B, alpha, beta based on N0, kel, kp, kd

alpha = 0.5 * (tmp1 + sqrt(tmp1^2 - 4*tmp2));

beta = 0.5 * (tmp1 - sqrt(tmp1^2 - 4*tmp2));

A = (kp+kel-beta)*N0/(alpha-beta);

B = (kp+kel-alpha)*N0/(beta-alpha);

fprintf('---------------------------------------------------------\n');

fprintf('Calculated: A(mg)=%d, B(mg)=%d, alpha(h^-1)=%d, beta(h^-1)=%d\n', A,B,alpha,beta);

break;

else

fprintf('Only a or b should be entered. Start over...\n\n');

continue;

end

end

%% Step 2: specify the rate constants kepr and kb

s2 = 'y';

while(s2 ~= 'n')

fprintf('---Step 2: enter kepr and kb ---\n');

kepr = input('kepr(h^-1) = ');

kb = input('kb(h^-1) = ');

T = input('Time(h) = ');

fprintf('You entered: kepr(h^-1)=%d, kb(h^-1)=%d, T(h)=%d\n', kepr,kb, T);

filename = input('Please input filename for simulation: ', 's');

% run the differential equation solver in MATLAB

Rate = [kp, kd, kel, kepr, kb];

[Tn,Y] = ode45(@ode_solver, [0 T], [N0 0 0 0], [], Rate);

% ode45 is a standard subroutine in MATLAB for solving differential

% equations

Nn = Y(:,1); % drug amount in blood

Nt = Y(:,3); % drug amount in tumor

% plot drug amount in blood vs. time

figure(1);

semilogy(Tn, Nn, 'r-', 'LineWidth', 2);

xlabel('Time (hours)','FontSize', 16);

ylabel('Nbl (mg)','FontSize', 16);

title('Drug in blood vs. Time','FontSize', 16);

saveas(1, [ResFolder, '\', filename, '_blood.tif']);

% plot drug amount in tumor vs. time

figure(2);

semilogy(Tn, Nt, 'r-', 'LineWidth', 2);

xlabel('Time (hours)','FontSize', 16);

ylabel('Nt (mg)','FontSize', 16);

title('Drug in tumor vs. Time','FontSize', 16);

saveas(2, [ResFolder, '\', filename, '_tumor.tif']);

% save the simulation parameters and results in .csv file

fid = fopen([ResFolder, '\', filename, '_SimRes.csv'], 'w');

fprintf(fid, 'Simulation parameters\n');

fprintf(fid, 'A(mg),B(mg),alpha(h^-1),beta(h^-1)\n');

fprintf(fid, '%d,%d,%d,%d\n',A,B,alpha,beta);

fprintf(fid, 'N0(mg),kel(h^-1),kp(h^-1),kd(h^-1)\n');

fprintf(fid, '%d,%d,%d,%d\n',N0,kel,kp,kd);

fprintf(fid, 'kepr(h^-1),kb(h^-1),T(h),FileName\n');

fprintf(fid, '%d,%d,%d,%s\n\n',kepr,kb,T,filename);

fprintf(fid, 'Simulation results\n');

fprintf(fid, 'Time(h),Nn(mg),Nt(mg)\n');

for i = 1:length(Tn),

fprintf(fid, '%d,%d,%d\n', Tn(i),Nn(i),Nt(i));

end

fclose(fid);

fprintf('*************************Step2 finished************************\n\n');

s2 = input('Would you like to continue with another kepr and kb? y/n: ', 's');

fprintf('\n');

end

fprintf('EPR finished.\n');

end

% ode_solver is customized ordinary differential equation solver called by

% standard subroutine in MatLab, i.e. ode45

function dy = ode_solver(t, y, Rate) %#ok<INUSL>

kp = Rate(1); kd = Rate(2); kel = Rate(3); kepr = Rate(4); kb = Rate(5);

dy = zeros(4,1);

dy(1) = -(kp + kel + kepr) * y(1) + kd * y(2) + kb*y(3);

dy(2) = kp * y(1) - kd * y(2);

dy(3) = kepr * y(1) - kb * y(3);

dy(4) = kel * y(1);

end
