# Supplementary material for: Quantitative Analysis of the Enhanced Permeation and Retention (EPR) Effect
Source: PLoS One. 2015 May 4;10(5):e0123461. doi: 10.1371/journal.pone.0123461 (PMC4418820; doi:10.1371/journal.pone.0123461)
Supplement: S1 Table — (DOCX) [file pone.0123461.s003.docx]

# The architecture of tumor vasculature

| **Cell Type** | **Location & host** | **Structure** | **Characteristic measurement** | **Method** | **Tumor size** | **Ref** |
| --- | --- | --- | --- | --- | --- | --- |
| Primary human colorectal adenocarcinoma | Human colon | Intervessel distance  tumor center  tumor surface  tumor periphery  Vessel diameter  tumor center  tumor surface  tumor periphery | 134.9 μm  89.1 μm  45.7 μm  22.9 μm  16.6 μm  18.2 μm | Corrosion casts, microCT & SEM | > 0.8 - 1.0 cm | [[1](#_ENREF_1)] |
| Human colorectal carcinomas (LS174T) | Flanks of nude (nu/nu, MF1) mice | Intervessel distance  Vessel diameter | 45.7 μm  18.2 μm | Corrosion casts, microCT & SEM | 0.88 ± 0.27 g | [[1](#_ENREF_1)] |
| Primary human colorectal cancer | Human colon | Intervessel distance  tumor center  tumor surface  tumor periphery  Vessel diameter  tumor center  tumor surface  tumor periphery | 177.7 μm  118.6 μm  54.3 μm  30.9 μm  18.3 μm  19.4 μm | Corrosion casts & SEM | > 0.8 - 1.0 cm | [[2](#_ENREF_2)] |
| Murine carcinoma (CaX) | Dorsal xenograft CBA/GyfTO mice | Intervessel distance  Vessel diameter | 112.4 µm  24.4 μm | Corrosion casts & SEM | 0.15 - 0.9 mL | [[3](#_ENREF_3)] |
| Murine carcinoma (CaNT) | Dorsal xenograft CBA/GyfTO mice | Intervessel distance  Vessel diameter | 213.6 µm  24.3μm | Corrosion casts & SEM | 0.15 - 0.9 mL | [[3](#_ENREF_3)] |
| Murine sarcoma (SaS) | Dorsal xenograft CBA/GyfTO mice | Intervessel distance  Vessel diameter | 180.7 µm  32.0 μm | Corrosion casts & SEM | 0.15 - 0.9 mL | [[3](#_ENREF_3)] |
| LHuman adenocarcinoma (HEC-1B) | Foreleg xenograft NCr-nu/nu mice | Intervessel distance  Vessel diameter | 76.1 µm  17.5 | Corrosion casts & SEM | 0.15 - 0.9 mL | [[3](#_ENREF_3)] |
| Rat mammary adenocarcinoma (R3230AC s.c.) | Ovarian fat pad of Fischer 344 rats | Intercapillary distance  Capillary diameter  Capillary length | 49 µm  10 μm  67 μm | Corrosion casts & SEM | 4 -5 g | [[4](#_ENREF_4)] |
| Human metastatic prostate cancer | Human prostate | Mean microvessel count | 104/mm^2^ | IHC | Not measured | [[5](#_ENREF_5)] |
| Human metastatic breast cancer | Human breast tissue | Mean microvessel count | > 140/mm^2^ | IHC | Not measured | [[6](#_ENREF_6)] |
| Mouse mammary sarcoma (EMT6) | Right flank of BALB/c mice | Blood vessel density | 14% | IHC | 8 - 12 mm diameter | [[7](#_ENREF_7)] |
| Human prostatic carcinoma (PC-3) | Right flank of athymic nude male mice | Blood vessel density | 16% | IHC | 8 - 12 mm diameter | [[7](#_ENREF_7)] |
| Mouse mammary adenocarcinoma (16C) | C3H mice | Blood vessel density | 14% | IHC | 8 - 12 mm diameter | [[7](#_ENREF_7)] |
| Murine mammary carcinoma (C3H/Bi) | Mouse rear leg gastrocnemius muscle | Vascular volume  Vascular surface area*  Vessel length* | 16.8%  < 15 mm^2^/mm^3^  < 150 mm/mm^3^ | Chalkley counts | 35 - 1500 mm^3^  > 500 mm^3^ | [[8](#_ENREF_8)] |
| Murine mammary adenocarcinoma (72j) | Subcutaneous axillary region of C3H mice | Vascular volume  Vascular surface area**  Vascular length** | 17%  < 11 mm^2^/mm^3^  < 100 mm/mm^3^ | Chalkley counts | 30 - 2410 mg  350 - 2700 mg | [[9](#_ENREF_9)] |

# S1 Table. Summary of parameters defining vasculature architecture in xenografts and human tumors. IHC – immunohistochemistry.

1. Folarin AA, Konerding MA, Timonen J, Nagl S, Pedley RB (2010) Three-dimensional analysis of tumour vascular corrosion casts using stereoimaging and micro-computed tomography. Microvasc Res 80: 89-98.

2. Konerding MA, Fait E, Gaumann A (2001) 3D microvascular architecture of pre-cancerous lesions and invasive carcinomas of the colon. Br J Cancer 84: 1354-1362.

3. Konerding MA, Malkusch W, Klapthor B, van Ackern C, Fait E, et al. (1999) Evidence for characteristic vascular patterns in solid tumours: quantitative studies using corrosion casts. Br J Cancer 80: 724-732.

4. Less JR, Skalak TC, Sevick EM, Jain RK (1991) Microvascular architecture in a mammary carcinoma: branching patterns and vessel dimensions. Cancer Res 51: 265-273.

5. Weidner N, Carroll PR, Flax J, Blumenfeld W, Folkman J (1993) Tumor angiogenesis correlates with metastasis in invasive prostate carcinoma. Am J Pathol 143: 401-409.

6. Horak ER, Leek R, Klenk N, LeJeune S, Smith K, et al. (1992) Angiogenesis, assessed by platelet/endothelial cell adhesion molecule antibodies, as indicator of node metastases and survival in breast cancer. Lancet 340: 1120-1124.

7. Primeau AJ, Rendon A, Hedley D, Lilge L, Tannock IF (2005) The distribution of the anticancer drug Doxorubicin in relation to blood vessels in solid tumors. Clin Cancer Res 11: 8782-8788.

8. Hilmas DE, Gillette EL (1974) Morphometric analyses of the microvasculature of tumors during growth and after x-irradiation. Cancer 33: 103-110.

9. Vogel AW (1965) Intratumoral Vascular Changes with Increased Size of a Mammary Adenocarcinoma: New Method and Results. J Natl Cancer Inst 34: 571-578.
